# Six year follow-up of students who participated in a school-based physical activity intervention: a longitudinal cohort study

**DOI:** 10.1186/1479-5868-6-48

**Published:** 2009-07-29

**Authors:** Lisa M Barnett, Eric van Beurden, Philip J Morgan, Lyndon O Brooks, Avigdor Zask, John R Beard

**Affiliations:** 1 Faculty of Health, Medicine, Nursing and Behavioural Sciences, Deakin University, Burwood Highway, Melbourne, Australia; 2 Health Promotion Unit, North Coast Area Health Service, Lismore, NSW Australia; 3 Faculty of Education and Arts, University of Newcastle, Newcastle NSW Australia; 4 Graduate Research College, Southern Cross University, Lismore, NSW Australia; 5 New York Academy of Medicine, 1216 Fifth Ave, New York, New York, USA

## Abstract

**Background:**

The purpose of this paper was to evaluate the long-term impact of a childhood motor skill intervention on adolescent motor skills and physical activity.

**Methods:**

In 2006, we undertook a follow-up of motor skill proficiency (catch, kick, throw, vertical jump, side gallop) and physical activity in adolescents who had participated in a one-year primary school intervention *Move It Groove It (MIGI) *in 2000. Logistic regression models were analysed for each skill to determine whether the probability of children in the intervention group achieving mastery or near mastery was either maintained or had increased in subsequent years, relative to controls. In these models the main predictor variable was intervention status, with adjustment for gender, grade, and skill level in 2000. A general linear model, controlling for gender and grade, examined whether former intervention students spent more time in moderate-to-vigorous physical activity at follow-up than control students.

**Results:**

Half (52%, *n *= 481) of the 928 MIGI participants were located in 28 schools, with 276 (57%) assessed. 52% were female, 58% in Grade 10, 40% in Grade 11 and 54% were former intervention students. At follow-up, intervention students had improved their catch ability relative to controls and were five times more likely to be able to catch: OR_catch _= 5.51, CI (1.95 – 15.55), but had lost their advantage in the throw and kick: OR_throw _= .43, CI (.23 – .82), OR_kick _= .39, CI (.20 – .78). For the other skills, intervention students appeared to maintain their advantage: OR_jump _= 1.14, CI (.56 – 2.34), OR_gallop _= 1.24, CI (.55 – 2.79). Intervention students were no more active at follow-up.

**Conclusion:**

Six years after the 12-month MIGI intervention, whilst intervention students had increased their advantage relative to controls in one skill, and appeared to maintain their advantage in two, they lost their advantage in two skills and were no more active than controls at follow up. More longitudinal research is needed to explore whether gains in motor skill proficiency in children can be sustained and to determine the intervention characteristics that translate to subsequent physical activity.

## Background

Regular participation in physical activity is associated with important health benefits for youth [1,2]. Physical activity in adolescence is also associated with subsequent adult activity levels, suggesting lifelong benefits [2,3]. The ability to perform fundamental motor skills, such as jumping, throwing or kicking has been positively associated with physical activity participation in both childhood [4] and adolescence [5], and is considered an important prerequisite to sport participation [6,7]. Fundamental motor skills are generally developed during childhood [7,8], and it has also been suggested that being motor skilled in childhood may have subsequent benefits on skill and activity levels in adolescence [9,10].

However, only limited research has been undertaken to determine how motor skill proficiency can be improved in children without developmental delay. This research indicates that motor skill improvement in a range of skills and populations is possible beyond that expected through normal growth and development [11-15]. In 2000, Move It Groove It (MIGI), a one-year school-based intervention in New South Wales (NSW), Australia, increased children's overall motor skill proficiency by 17% [11]. Another more recent Australian one-year intervention, 'Switch-Play', amongst other goals, aimed to: improve fundamental motor skills and prevent declines in physical activity. Children in Grade 5 in primary school (10–11 years old) had their school class randomized to one of three intervention groups: i) behavior modification, ii) fundamental motor skills, and iii) both behavior modification and fundamental motor skills, or a control group (usual curriculum). Whilst no significant intervention effects for fundamental motor skill z-scores were found overall, girls in two of the intervention groups, i) behavior modification and ii) fundamental motor skills, recorded significantly higher motor skill z-scores at post intervention [15]. The Children's Health InterventionaL Trial (CHILT), a four-year German primary prevention program to combat overweight and obesity in childhood also included a motor skill component. Children commenced the intervention in their first year at school and after an intermediate assessment at 20 months, lateral jumping ability was found to be greater in the intervention group [14]. After the intervention there was also significant improvement in two of the four motor items assessed – balancing backwards and lateral jumping [13]. Furthermore, British children who participated in a nine week after-school multiskills club performed significantly better in the static balance following the intervention [16]. Earlier, Halverson and Roberton demonstrated the positive effects of instruction on throwing ability in American children [12].

While interventions to improve motor skill levels in childhood thus appear to hold some promise, less is known about their long-term impact. To address this gap in the literature, we re-assessed MIGI participants six years after the intervention was completed, to (i) determine whether the probability of children in the intervention group being able to perform each skill was either maintained or had increased in the subsequent years, relative to controls, and (ii) whether the skill gains intervention students experienced in childhood led to greater physical activity participation as adolescents.

## Methods

### Move It Groove It

MIGI was a one-year intervention which aimed to increase motor skill proficiency and physical activity amongst primary school students [11] with the expectation that intervention children would become more involved in sports, and remain more active than their lesser skilled peers [10]. The design was quasi-experimental with interested schools in a 24,555 sq km area in New South Wales, Australia, randomly selected and stratified by size and district. Nine schools participated in the in-school intervention and nine served as controls. Motor skill ability of 1045 children was assessed between February and June 1999 (MIGI baseline) and August to December 2000 (MIGI post-test) [11]. A total of 1045 students were assessed at MIGI post-test, 53% boys and 47% girls with the mean age of the sample, 10.1 years (range 7.9 to 11.9).

### The Physical Activity and Skills Study (PASS)

The PASS was a six-year follow-up of MIGI. Of the 1045 students assessed for MIGI, 928 eligible students were matched by name and gender to the class roll. During 2006, this list of MIGI participants was sent to 41 consenting high schools in the original study region to locate participants for follow-up. One school did not consent. Students identified on the high school register were invited to participate, and those who returned a consent form signed by parents/guardian and themselves to the nominated school contact were included in the PASS.

### Data Collection

Data for the current study were collected by the study coordinator and three research assistants trained in motor skill assessment and survey administration. Over 94% of data were collected over Term Four in 2006, with the remainder in Term One, 2007; both over what are known in Australia as the summer school terms. Ethics approval was gained from the relevant bodies.

### Motor skill measurement

In both the original (MIGI) and current study (PASS), the Australian resource, 'Get Skilled Get Active' [17] was used to assess motor skills. In MIGI, eight skills (catch, overhand throw, kick, vertical jump, side gallop, hop, sprint run and static balance) were assessed with interrater reliability, kappa = .61 [11]. In the PASS, three object control skills (manipulation of an object); kick, catch and throw, and three locomotor skills; hop, side gallop and vertical jump, were reassessed, with interrater reliability, kappa = .70 [18].

Each skill has a number of features considered integral to the proficient performance of the skill. The kick, catch, overhand throw, and vertical jump all have six features whilst the hop and side gallop have five features. For example, the five features of the side gallop are: 1. *Smooth rhythmical movement*. 2. *Brief period where both feet are on the ground*. 3. *Weight on the balls of the feet*. 4. *Hips and shoulders point to the front*. 5. *Head stable, eyes focused forward or in direction of travel*.

Testing procedure allowed students to observe a skill demonstration before being asked to perform the skill [17]. Most skills were performed five times, but the hop and side gallop were observed as students traveled back and forth once between two points 15 meters apart. Each feature of each skill was assessed as present or absent without any verbal feedback from the assessor. Assessment was completed by observing critical features in the order in which they are executed. For instance with the catch 'eyes focused' is the preparatory feature, followed by 'feet moving to place the body in line with the object', then 'hands coming to meet the object' etc. Whilst observing critical features, if the assessor noticed a feature they had previously checked as present was not performed correctly in a subsequent trial, they would revert back to that feature and watch again in the next trial to see whether it was present or absent. If the assessor was at all unsure, they could ask the student to perform the skill again and if the feature was performed fairly consistently over all the trials, it was checked as present. However, if there was any uncertainty about whether a feature was consistently present or not, the assessor was instructed to check the feature as absent [18].

### Physical Activity Measurement

The Adolescent Physical Activity Recall Questionnaire (APARQ) measured physical activity participation [19]. APARQ has moderate test-retest reliability and validity [19] and has been used in numerous other studies [5,20-23]. Students specified all organised and non-organised physical activities in which they participated in during a usual week, in both summer and winter terms, and frequency and duration of participation. Students also indicated date of birth, school grade, gender, Aboriginal and/or Torres Strait Islander status and language spoken at home.

### Data Management and Analysis

Students recruited for the PASS had their childhood MIGI assessments, conducted in 2000 at MIGI post-test [11], reanalysed and reported for use in the current study. Thus the PASS reports two time points: childhood post intervention (2000) and adolescence (2006/07). All analysis used SPSS version 15.0 (SPSS Inc. ). We were unable to link to the baseline MIGI (1999) motor skill scores because the initial MIGI ethics approval did not enable us to identify students at baseline. At MIGI post-test, initials were stored with students FMS scores and the ethics approval for the PASS enabled us to use this data to match with student roll information in order to identify students for follow up.

For the motor skill scores, the number of features rated correct for each skill as performed in childhood and adolescence was summed for each participant. A binary variable Mastery/Near Mastery (MNM) was created for each skill for students who had achieved mastery (all features correct) or near mastery (only one feature incorrect), compared to those who had more than one feature incorrect [11].

Each physical activity was assigned a MET (metabolic equivalent, 1 MET = 3.5 mL of oxygen per kilogram of body weight per minute) from a comprehensive list of physical activities [24], since expanded [21]. Activities < 10 minutes in duration or < once per week were excluded, as was light activity; MET value of < 3.0 [21]. Total time in moderate-to-vigorous physical activity (MVPA) per week in minutes was averaged between seasons and log transformed prior to analysis to normalize distribution. Three cases were excluded as they had reported a nil physical activity value therefore creating a skewed dataset.

Chi Square tests were used to determine intervention/control differences in performance of each skill to MNM level at post intervention. A Bonferroni adjustment for multiple comparisons (calculated by dividing the number of tests – six in each age period – by the determined alpha of .05) was used, with the corrected alpha, *p *< .0083. A series of logistic regression models assessed for each skill (except the hop) whether the probability of children in the intervention group achieving MNM was either maintained or had increased in the subsequent years, relative to controls. Only the skills in which intervention children performed better than controls at post-test (even if not reaching significance) were assessed; therefore the hop was excluded.

In each model, the outcome variable was the ability to perform the respective skill to a MNM level, the main predictor variable was intervention status, controlling for gender, grade and ability to perform the skill at post-test. Non-significant main effects were retained to provide a basis to compare the skills. Inclusion of post MIGI intervention MNM status in the logistic regression models means that the intervention effect estimates the relative change in the probability of MNM between the groups since the intervention. For skills that had shown a significant improvement due to MIGI, an odds ratio ≥ 1.0 for the intervention effect in the model indicates that the positive effect of the intervention was maintained or had increased over time, while an odds ratio < 1.0 indicates that the intervention group advantage had decreased.

A general linear model was conducted to examine the relationship between being an intervention or control student and the dependent variable: weekly minutes spent in MVPA. This model included the intervention/control variable, and controlled for grade and gender, in order to examine whether the effect on physical activity in adolescence differed for intervention or control students. The model also included corresponding two-way interactions involving the intervention/control variable. Ability to perform each skill to a MNM level at post-test was not adjusted for as this would have effectively removed the advantage the intervention students had from the MIGI intervention. Non-significant variables and interactions were removed, except for the intervention/control variable as it was the variable of interest. An alpha of .05 was used to determine significance for all models.

## Results

### Sample

**Figure 1:**
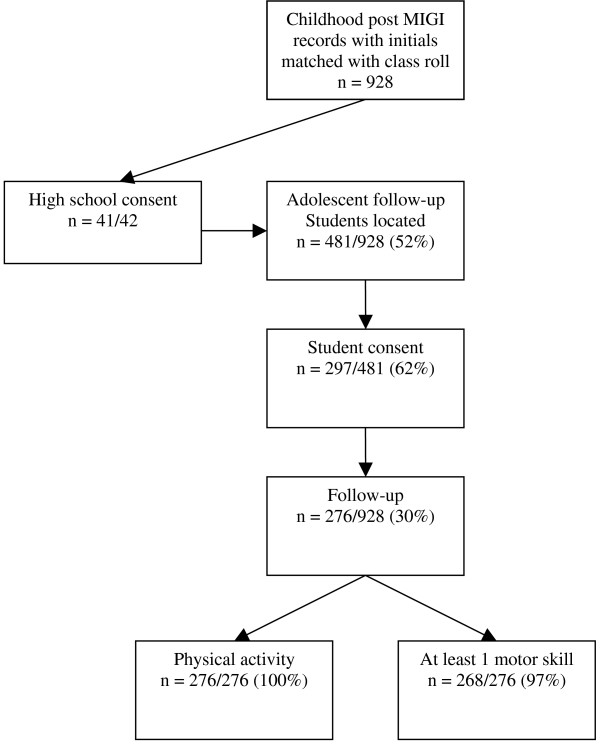
**Flow chart of consent and follow up numbers in the PASS**.

Figure 1 provides a flow chart of the consent and follow-up rates. Intervention students were more likely to consent (*n *= 156/219) than controls (*n *= 140/262) (*χ*^2 ^= 15.97, *p *< .000). The followed up sample did not differ from the overall cohort by gender (*χ*^2 ^= 2.40, *p *= .12) but were more likely to have been originally tested in Grade 4 (61.5%) than Grade 5 (38.5%), (*χ*^2 ^= 22.67, *p *< .0001) and had a higher mean composite childhood fundamental motor skill score; 17.5 compared to 16.5, (*t *= -2.60, *p *= 0.009).

Of the 276 students in the current study, all students completed the APARQ, and 268 were assessed for at least one motor skill. More were in Grade 10 (58.2%, *n *= 160/275) with 41.8% (*n *= 115) in Grade 11. Mean age was 16.4 (range 14.2 to 18.3 yrs), 52.2% were female (*n *= 144/276) and 53.6% (*n *= 148) intervention students. All but one spoke English at home and 7.0% (*n *= 19/271) identified as Aboriginal or Torres Strait Islander.

### Motor skill proficiency: comparison of intervention and control students

At post intervention, intervention students were significantly better at performing the kick but not the other five skills, see Table 1. At follow-up, intervention students had improved their catch ability relative to controls and were five times more likely to be able to catch: OR_catch _= 5.51, CI (1.95 – 15.55). Intervention students had lost their advantage in the overhand throw: OR_throw _= .43, CI (.23 – .82), and kick: OR_kick _= .39, CI (.20 – .78). For the other skills, intervention students appeared to maintain their advantage: OR_jump _= 1.14, CI (56 – 2.34), OR_gallop _= 1.24, CI (.55 – 2.79). See Table 2.

**Table 1:** Percentage of intervention/control students who reached mastery/near mastery level at post intervention (2000) by skill

Skills	Childhood					
	
	% mastery/near mastery level				I/C differences	
	
	N	Control	N	Intervention	Chi Square	*p*
Catch	127	45.7	148	59.5	5.22	.022
Kick	127	25.2	148	41.9	8.47	.004*
Overhand Throw	125	22.4	147	36.7	6.59	.010
Side Gallop	127	43.3	146	52.1	2.08	.149
Vertical Jump	125	24.8	143	33.6	2.47	.116
Hop	127	15.9	147	13.5	0.30	.581

Note: Different Ns due to missing data I/C = Intervention/Control

α = .0083 (Bonferroni corrected adjustment)

**Table 2:** Logistic regression models showing whether intervention students maintained or increased their advantage, relative to controls in terms of performance of each skill to mastery/near mastery level (MNM) at follow-up; controlling for MNM level at end of intervention, gender and grade

Skills							95% CI	
	
		Beta	SE	Wald	P	Odds ratio	Lower	Upper
Catch	Intercept	1.80	.57	9.86	.002			
	MNM in 2000	.40	.45	.77	.380	1.49	.61	3.61
	Grade	-1.23	.54	5.24	.022	.29	.10	.84
	Gender	.97	.48	4.01	.045	2.63	1.02	6.78
	Intervention status	1.71	.53	10.39	**.001**	**5.51**	1.95	15.55

Throw	Intercept	2.36	.42	31.92	.000			
	MNM in 2000	.47	.39	1.47	.226	1.60	.75	3.41
	Grade	-2.13	.36	34.27	.000	.12	.06	.24
	Gender	-.21	.32	.00	.948	.98	.53	1.83
	Intervention status	-.84	.33	6.57	**.010**	**.43**	.23	.82

Kick	Intercept	1.86	.42	19.40	.000			
	MNM in 2000	.66	.37	3.07	.080	1.93	.93	4.00
	Grade	-2.69	.37	52.78	.000	.07	.03	.14
	Gender	.09	.32	.07	.787	1.09	.58	2.06
	Intervention status	-.93	.35	7.05	**.008**	**.39**	.20	.78

Jump	Intercept	1.07	.37	8.19	.004			
	MNM in 2000	1.19	.51	5.51	.019	3.27	1.22	8.81
	Grade	.27	.36	.56	.456	1.31	.64	2.68
	Gender	.33	.36	.82	.364	1.39	.68	2.84
	Intervention status	.14	.36	.14	**.712**	**1.14**	.56	2.34

Gallop	Intercept	2.32	.48	23.29	.000			
	MNM in 2000	.04	.41	.01	.932	1.04	.46	2.31
	Grade	-.50	.43	1.37	.242	.61	.26	1.40
	Gender	-.20	.41	.24	.623	.82	.37	1.82
	Intervention status	.21	.42	.26	**.613**	**1.24**	.55	2.79

Note: Reference categories were: 'Male', 'Yr 10', 'Control' and '0 Skill'

### Physical activity participation: comparison of intervention and control students

Overall mean time in MVPA at follow-up was 826 minutes per week (*SD *= 551.1) with no intervention and control differences (*t *= -0.66, *p *= 0.51). Being an intervention or control student did not have a significant effect on MVPA (β = -.09, CI -.26 – .09), see Table 3. All interactions involving the intervention/control variable were non-significant and removed.

**Table 3:** General linear model testing childhood intervention effect on weekly MVPA minutes in adolescence

	Final Model			
Effect	Beta	p	Lower CI	Upper CI
Intercept	6.08	.000	5.89	6.27
Gender (Male = 0, Female = 1)	-.42	.000	-.25	-.59
Grade (Grade 10 = 0, Grade 11 = 1)	-.27	.002	-.10	-.45
Intervention status (Control = 0, Intervention = 1)	-.09	.318	-.26	.09

Model – R Squared = .104 (Adjusted R Squared = .094)

MVPA = moderate-to-vigorous physical activity

Note: 95% used for confidence intervals.

## Discussion

To our knowledge, this is the first study that has investigated the long-term impact of a primary school-based motor skill intervention. Of the five skills assessed for follow-up differences, intervention students had increased their advantage relative to controls in the catch, lost their advantage in the kick and the overhand throw and appeared to maintain their advantage in the side gallop and jump. Being an intervention student did not result in higher adolescent MVPA participation at follow-up.

Our mixed results for individual motor skills can perhaps be explained by the fact that there was no MIGI follow-up program to maintain gains in intervention students. Also, in subsequent years many environmental factors such as parental or other support, sibling physical activity, participation in community sports, opportunities to exercise [25] and quality of experience in school physical education [26] may have influenced both control and intervention students' motor skills. Since motor skill development is based on an interaction between constraints from the task, organism and environment [27], motor skills can be influenced and improved at any point in development. It is also possible that intervention students may have lost their advantage in the kick and throw because control students had greater potential to change due to a possible ceiling effect operating within the instrument. For example, around 15% more intervention students had achieved mastery or near mastery in the kick and overhand throw at post intervention.

Unfortunately, there are no motor skill and very few school-based physical activity interventions with long-term follow-up to which we can compare our findings. Reviews of strategies to promote physical activity amongst young people recommend more long-term studies [28,29]. Likewise, many of the studies investigating the relationship between motor skill and physical activity suggest that future research include intervention studies [4,5,30-32].

The 'Switch-Play' intervention had a 12 month follow-up and found that girls in two of the intervention groups (behavior modification and fundamental motor skill), recorded significantly higher motor skill z-scores [15]. Also, significant positive average differences in movement counts per day and vigorous intensity physical activity were found for children in the behavior modification group and in the fundamental motor skills group compared to controls, with the greatest effects for those in the motor skill group [15].

Only three physical activity interventions with long-term follow-up were located: CATCH – three year follow-up [33], Class of 1989 – twelve years [34] and the Oslo Youth Study – seven years [28], all reporting significant intervention effects on self-reported physical activity that declined over time [28]. Three years after the CATCH study, intervention students still reported between 8.8 and 13.6 minutes more daily vigorous physical activity than controls [33]. MIGI differed to these studies in that MIGI examined physical activity in two different group contexts (physical education lessons and in the playground during break periods) which could not be linked by person or presented as total daily self-reported activity [33] or as hours of exercise per week [34]. MIGI had no effect on playground activity at post-test [35] and only a very small effect on physical activity in physical education lessons, with the gains in MVPA translating to less than one minute per average 21 minute physical education lesson [11]. It is highly unlikely that these negligible gains would contribute to future physical activity in intervention students, thus any expected physical activity effect after six years was anticipated to be as a result of motor skill improvement gained through MIGI.

Our study rationale for MIGI was based on motor skill learning theories e.g. Competence Motivation Theory [36] and physical activity behavior tracking [37]. Since MIGI, McKenzie and colleagues found no relationship between childhood movement skills (ages 4–6 yrs) and subsequent physical activity (12 yrs) [38]. A separate component of the PASS did find childhood skill predicted subsequent physical activity [39], but the current study has demonstrated this was not due to a difference between intervention and control students. Perhaps the MIGI effect on motor skills was inadequate to translate to subsequent physical activity for intervention students. Greater gains achieved through a more intensive, targeted or longer intervention may be needed to produce effects substantial enough to translate to physical activity participation. MIGI interventions included: 'buddying' of pre-service teachers with each of the schools for in-class work with teachers and students, professional development of classroom teachers, collaborative planning with the schools project team and resource allocation in the form of a web-site and funding for equipment purchase [11]. Whilst the MIGI intervention had an effect on motor skills at post-test for intervention students [11], it is unclear which components of the intervention were most effective or how intensive the intervention delivery was in each school. Thus it is hard to establish what intervention intensity may be needed to produce motor skill effects necessary to translate to subsequent physical activity behavior.

### Study strengths and limitations

The main strength of this study is that it is one of very few studies to track the impact of a childhood school-based physical activity intervention over time. Furthermore, it is the only known study that has looked at the long term impact of a motor skill intervention with a 6-year follow-up. A major limitation was that while we could track students by name from post-test (2000) to follow-up (2006/07), we could not individually link them back to their MIGI baseline (1999) scores. We were therefore unable to adjust for baseline differences. In MIGI, control students at baseline were more proficient in most skills, with differences adjusted for within the analysis [11]. When we used the PASS sample to see whether intervention and control students differed in skill proficiency at MIGI post-test, we found intervention students were only significantly more proficient than controls in the kick (although more intervention students could perform each skill except the hop). This suggests that either baseline differences, or the follow-up sample size, masked the MIGI intervention effects on motor skills.

Another study limitation was the low follow-up rate. This was unavoidable due to the length of the follow-up period and difficulties locating students who had moved out of the region. However, loss to follow-up is unlikely to have biased our findings substantially as there was only a slight difference (one point on 30 point scale) in mean composite childhood skill score between intervention and control students. Consent rate was higher than for the same age group in a similar study [21], but there was some bias in that intervention children were more likely to consent.

It is possible that intervention effects on motor skill proficiency were not identified because a ceiling effect operating within the motor skill instrument may have masked differences. Using self-report for physical activity is an important limitation, and in addition, maturation [40] and weight status [22] were not controlled for, both factors that can affect motor skill performance.

## Conclusion

Six years after a 12 month primary school-based intervention to improve motor skill proficiency and physical activity levels, intervention students had increased their advantage relative to controls in one skill, lost their advantage in two skills and appeared to maintain their advantage in another two. Intervention students were no more physically active at long term follow-up, perhaps signifying intervention effects were not enough to translate to adolescent physical activity behavior. More longitudinal research is needed to design effective primary school-based physical activity interventions, to explore whether gains in motor skill proficiency in healthy children can be sustained and to characterise the impact of early motor skill gains in terms of subsequent physical activity behavior.

## Competing interests

The authors declare that they have no competing interests.

## Authors' contributions

LMB conceived, designed and carried out the study, and drafted the manuscript. EvB, PJM, JRB, participated in the conception and design of the study, helped in statistical interpretation and helped to draft the manuscript. LOB advised the statistical analysis and helped draft the manuscript. AZ helped in statistical interpretation and to draft the manuscript. All authors read and approved the final manuscript.

## References

[B1] Bauman AE (2004). Updating the evidence that physical activity is good for health: An epidemiological review 2000–2003. J Sci Med Sport.

[B2] Hallal PC, Victora CG, Azevedo MR, Wells JCK (2006). Adolescent physical activity and health: A systematic review. Sports Med.

[B3] Malina RM (1996). Tracking of physical activity and physical fitness across the lifespan. Res Q Exerc Sport.

[B4] Wrotniak BH, Epstein LH, Dorn JM, Jones KE, Kondilis VA (2006). The relationship between motor proficiency and physical activity in children. Pediatrics.

[B5] Okely AD, Booth M, Patterson JW (2001). Relationship of physical activity to fundamental movement skills among adolescents. Med Sci Sports Exerc.

[B6] Corbin CB, Corbin CB (1980). Motor performance and physical fitness in adolescence. A Textbook of Motor Development.

[B7] Haubenstricker J, Seefeldt V (1986). Acquisition of motor skills during childhood. Physical Activity and Well-Being.

[B8] Clark JE, Metcalfe JS, Clark JE, Humphrey JH (2002). The mountain of motor development: A metaphor. Motor development Research and reviews.

[B9] Stodden DF, Goodway JD, Langendorfer SJ, Roberton MA, Rudisill ME, Garcia C, Garcia LE (2008). A developmental perspective on the role of motor skill competence in physical activity: An emergent relationship. Quest.

[B10] McKenzie TL, Alcaraz JE, Faucett FN, Sallis JF (1997). Effects of physical education program on children's manipulative skills. J Teach Phys Educ.

[B11] van Beurden E, Barnett LM, Zask A, Dietrich UC, Brooks LO, Beard J (2003). Can we skill and activate children through primary school physical education lessons? 'Move it Groove It' – a collaborative health promotion intervention. Prev Med.

[B12] Halverson LE, Roberton MA, Newell K, Roberts G (1978). The effects of instruction on overhand throwing development in children. Psychology of Motor Behavior and Sport.

[B13] Graf C, Koch B, Falkowski G, Jouck S, Christ H, Staudenmaier K, Tokarski W, Gerber A, Predel HG, Dordel S (2008). School-based prevention: Effects on obesity and physical performance after 4 years. J Sports Sci.

[B14] Graf C, Koch B, Falkowski G, Jouck S, Christ H, Staudenmaier K, Tokarski W, Gerber A, Predel HG, Dordel S (2005). Effects of a school based intervention on BMI and motor abilities in childhood. J Sport Sci Med.

[B15] Salmon J, Ball K, Hume C, Booth M, Crawford D (2008). Outcomes of a group-randomized trial to prevent excess weight gain, reduce screen behaviours and promote physical activity in 10-year-old children: Switch-Play. Int J Obes.

[B16] Foweather L, McWhannell N, Henaghan J, Lees A, Stratton G, Batterham AM (2008). Effect of a 9-wk. after-school multiskills club on fundamental movement skill proficiency in 8- to 9-yr.-old children: An exploratory trial. Percept Mot Skills.

[B17] New South Wales Department of Education and Training (2000). Get Skilled: Get Active.

[B18] Barnett LM, van Beurden E, Morgan PJ, Lincoln D, Zask A, Beard JR (2009). Interrater objectivity for field-based fundamental motor skill assessment. Research Quarterly for Exercise Science and Sport 80.

[B19] Booth M, Okely AD, Chey T, Bauman A (2002). The reliability and validity of the Adolescent Physical Activity Recall Questionnaire. Med Sci Sports Exerc.

[B20] Booth M, Macaskill P, McLellan L, McLellan L, Okely T (1997). NSW schools fitness and physical activity survey 1997.

[B21] Booth M, Okely AD, DenneyWilson E, Hardy L, Yang B, Dobbins T (2006). NSW Schools Physical Activity and Nutrition Survey (SPANS) 2004 Full Report.

[B22] Okely AD, Booth M, Chey T (2004). Relationship between body composition and fundamental movement skills among children and adolescents. Res Q Exerc Sport.

[B23] Okely AD, Booth M (2004). Mastery of fundamental movement skills among children in New South Wales: prevalence and sociodemographic distribution. Journal of Science & Medicine in Sport.

[B24] Ainsworth BE, Jacobs DR, Leon AS (1993). Compendium of physical activities: Classification of energy costs of human physical activities. Med Sci Sports Exerc.

[B25] Sallis JF, Prochaska JJ, Taylor WC (2000). A review of correlates of physical activity of children and adolescents. Med Sci Sports Exerc.

[B26] Morgan PJ, Hansen V (2008). The relationship between PE biographies and PE teaching practices of classroom teachers. Sport, Education and Society.

[B27] Newell K, Wade MG, Whiting HT (1986). Constraints on the development of coordination. Motor Development in Children: Aspects of Coordination and Control.

[B28] Stone EJ, McKenzie TL, Welk GJ, Booth ML (1998). Effects of physical activity interventions in youth: Review and synthesis. Am J Prev Med.

[B29] Timperio A, Salmon J, Ball K (2004). Evidence-based strategies to promote physical activity among children, adolescents and young adults: Review and update. J Sci Med Sport.

[B30] Butcher JE, Eaton WO (1989). Gross and fine motor proficiency in preschoolers: Relationships with free play behavior and activity level. J Hum Move Studies.

[B31] Fisher A, Reilly JJ, Kelly LA, Montgomery C, Williamson A, Paton J (2005). Fundamental movement skills and habitual physical activity in young children. Med Sci Sports Exerc.

[B32] Raudsepp L, Pall P (2006). The relationship between fundamental motor skills and outside-school physical activity of elementary school children. Pediatr Ex Sci.

[B33] Nader PR, Stone EJ, Lytle LA, Perry CL, Osganian SK, Kelder S, Webber LS, Elder JP, Montgomery D, Feldman HA, Wu M, Johnson C, Parcel GS, Luepker RV (1999). Three-year maintenance of improved diet and physical activity: The CATCH cohort. Arch Pediatr Adolesc Med.

[B34] Kelder SH, Perry CL, Klepp KI (1993). Community-wide youth exercise promotion: Long-term outcomes of the Minnesota Heart Health program and the Class of 1989 study. J Sch Health.

[B35] Zask A, van Beurden E, Barnett LM, Brooks LO, Dietrich UC (2001). Active School Playgrounds – Myth or Reality? Results of the "Move It Groove It" Project. Prev Med.

[B36] Harter S (1978). Effectance motivation reconsidered: Toward a developmental model. Hum Dev.

[B37] Telama R, Yang X, Viikari J, Valimaki I, Wanne O, Raitakari O (2005). Physical activity from childhood to adulthood: A 21-year tracking study. Am J Prev Med.

[B38] McKenzie TL, Sallis JF, Broyles SL, Zive MM, Nader PR (2002). Childhood movement skills: Predictors of physical activity in Anglo American and Mexican American adolescents?. Res Q Exerc Sport.

[B39] Barnett LM, van Beurden E, Morgan PJ, Brooks LO, Beard JR (2009). Childhood motor skill proficiency as a predictor of adolescent physical activity. J Adolesc Health.

[B40] Gabbard C (2008). Lifelong motor development.

